# Genome Sequence of *Kocuria palustris* Strain CD07_3 Isolated from the Duodenal Mucosa of a Celiac Disease Patient

**DOI:** 10.1128/genomeA.00210-16

**Published:** 2016-04-28

**Authors:** Atul Munish Chander, Ramesan Girish Nair, Gurwinder Kaur, Rakesh Kochhar, Shanmugam Mayilraj, Devinder Kumar Dhawan, Sanjay Kumar Bhadada

**Affiliations:** a Department of Biophysics, Panjab University, Chandigarh, India; b Microbial Type Culture Collection and Gene bank (MTCC), CSIR-Institute of Microbial Technology, Chandigarh, India; c Department of Gastroenterology, Postgraduate Institute of Medical Education and Research, Chandigarh, India; d Department of Endocrinology, Postgraduate Institute of Medical Education and Research, Chandigarh, India

## Abstract

We report here the 2.8-Mb genome of *Kocuria palustris* strain CD07_3 isolated from the duodenal mucosa of a celiac disease (CD) patient. The genome of the bacterium consists of specific virulence factor genes and antibiotic resistance genes that depict its pathogenic potential.

## GENOME ANNOUNCEMENT

Intestinal microbes are important in health and disease (1). Imbalanced intestinal microbial structure and some microbes are associated with celiac disease (CD) (2–4). CD is an autoimmune disorder of the small intestine in which certain pathogenic microbes modulate the disease presentation (5), suggesting the importance of each pathogenic microbe in disease. *Kocuria palustris* has rarely been reported in the literature. Although *K. palustris* DSM 11925^T^ caused unusual corneal infection early in the course of peripheral ulcerative keratitis in a patient (6), this is the first time to our knowledge that this microbe has been reported from a CD patient. In this instance, whole-genome sequencing of microbes can provide better insight into annotations for predicting their role in disease outcome (7). Here, we report the draft genome of the *K. palustris* strain CD07_3 isolated from the duodenal mucosa of a CD patient and specific annotations that reveal its pathogenic potential.

We report here the draft genome sequence of the *K. palustris* strain CD07_3 isolated from the duodenal mucosa of a CD patient of the Postgraduate Institute of Medical Education and Research (PGIMER), Chandigarh, India. The study was approved by the ethics committee of PGIMER, and a written consent was obtained from the participant. The subject was tTG IgA-antibody (Ab) positive (128 U/ml) and presented with dyspepsia, fatigability, short stature, and body aches.

Genomic DNA was extracted from a 48-h-old culture using the ZR fungal/bacterial DNA MiniPrep, as per the manufacturer’s instructions. The genome of *K. palustris* strain CD07_3 was sequenced using the Illumina-HiSeq 1000 technology. A total of 13,272,656 reads were generated, amounting to 1,278,722,539 bp, and were *de novo* assembled into 18 contigs using CLC Genomics Workbench version 7.5.1 (CLC bio, Aarhus, Denmark), with a total length of 2,833,422 bp and mean coverage of 100×. The assembly has an *N*_50_ of 416,347 bp and average contig length of 157,412 bp, with a mean G+C content of 70.4%. The functional annotation was carried out by Rapid Annotations using Subsystems Technology (RAST) (8). tRNA was predicted by ARAGORN (9) and rRNA genes by RNAmmer 1.2 (10). The genome contains a predicted total of 2,509 coding sequences (CDSs), 3 rRNAs, and 50 tRNAs.

Whole-genome annotation available at the RAST server shows that *K. palustris* strain CD07_3 contains genes of vancomycin B-type resistance protein VanW, DNA helicase, phage-associated genes, an AMP-dependent synthetase/ligase in alkaline synthesis cluster, excinuclease ABC subunit A, uracil-DNA glycosylase, family 1, cell division inhibitor, maltose/maltodextrin ABC transporter, substrate binding periplasmic protein MalE, and glutamine synthetase type 1 (EC 6.3.1.2). The functional comparison of genome sequences available on the RAST server revealed the closest neighbors of *K. palustris* strain CD07_3 to be *Kocuria rhizophila* DC2201 (score, 516), followed by *K. rhizophila* P7-4 (score, 419), *Arthrobacter* sp. strain FB24 (score, 386), and *Arthrobacter chlorophenolicus* A6 (score, 353).

### Nucleotide sequence accession numbers.

This whole-genome shotgun project has been deposited at DDBJ/EMBL/GenBank under the accession no. LQBJ00000000. The version described in this paper is the first version, LQBJ01000000.
